# Quantification of Antisense Oligonucleotides by Splint Ligation and Quantitative Polymerase Chain Reaction

**DOI:** 10.1089/nat.2021.0040

**Published:** 2022-01-31

**Authors:** Minwook Shin, Pranathi Meda Krishnamurthy, Gitali Devi, Jonathan K. Watts

**Affiliations:** RNA Therapeutics Institute, UMass Chan Medical School, Worcester, Massachusetts, USA.

**Keywords:** antisense oligonucleotides, quantification, SplintR ligase, qPCR

## Abstract

Reliable detection and quantification of antisense oligonucleotides (ASOs) in experimental and clinical specimens are essential to understand the biological function of novel oligonucleotide-based therapeutics. In this study, we describe a method to detect and quantify ASOs in biological samples, whereby the ASO acts as a splint to direct the ligation of complementary probes and quantitative real-time PCR was used to monitor ligation products. Low levels of 2′-*O*-methoxyethyl (2′-*O*-MOE) gapmer ASO in serum, liver, kidney, lung, heart, muscle, and brain tissues can be detected over a 6-log linear range for detection using this method. This method allows quantification of various types of chemically modified ASOs, including phosphorothioate linkage, 2′-*O*-methyl, 2′-*O*-MOE, and locked nucleic acid, as well as siRNAs. This method does not require probe modifications, and can be performed using standard laboratory equipment; making it a fast, sensitive, and reliable technique that can be widely applied. This detection method may find potential applications in detection of therapeutic oligonucleotides in biological samples.

## Introduction

Antisense oligonucleotides (ASOs) consist of DNA, RNA, or chemically modified nucleotides that regulate target RNA molecules through Watson-Crick base pairing [1–3]. For potential therapeutic applications, the physiological stability and pharmacokinetics of ASOs need to be optimized by introduction of chemical modifications to the phosphate linkage, ribose sugar, or nucleobase [4–6]. The phosphorothioate (PS) linkage involves the replacement of a nonbridging oxygen in the phosphodiester linkage with sulfur, which improves nuclease resistance and facilitates cell uptake through increased protein binding [7,8]. Modification of the ribose sugar with 2′-*O*-methyl (2′-OMe) RNA [9], 2′-*O*-methoxyethyl (2′-*O*-MOE) RNA [10], or locked nucleic acid (LNA) [11] improves affinity toward the target RNA, and increases the stability of the ASO by protecting it from nuclease digestion.

When fully modified ASOs tightly bind to target RNAs, they function as steric blockers to regulate functions such as splicing [12,13]. Gapmers are ASOs, which typically have a stretch of phosphorothioate-modified DNA in the central region to recruit RNase H, usually flanked by high-affinity, nuclease-stable modified nucleotides [9]. All of these modifications increase the potential use of ASOs as therapeutic agents in the treatment of life-threatening diseases. Novel chemical modifications, improved knowledge of mechanisms of action, and refined clinical trial designs have all contributed to increasing momentum for the translation of ASOs into the clinic [14]. Indeed, more than 300 ASOs are now in various stages of late preclinical and clinical development [15].

Simple and reliable bioanalytical methods to detect and quantify ASOs are essential to understand their mechanism of action and to transition research from preclinical to clinical settings [16]. The ASO detection methods being used currently vary in the costs incurred for sample preparation and analysis, as well as their sensitivity. Liquid chromatography coupled with mass spectrometry (LC-MS) can discriminate ASOs from their metabolites, but requires extensive sample processing, and the sensitivity of the method is relatively low, requiring ASO concentrations above 10 pM [17,18]. Hybridization-based enzyme-linked immunosorbent assays (ELISAs) have a higher sensitivity (100 fM) and can be performed with moderate throughput [19,20]; however, their linear range for detection is low, and the extensive sample processing required makes the approach time-consuming and expensive.

The quantitative real-time polymerase chain reaction (qPCR) has the highest sensitivity and broadest detection range. However, because of the short length of ASOs, it is rather challenging to apply qPCR to their quantitation [21]. Increasing the lengths of qPCR templates can address this limitation. For example, stem-loop reverse transcription (RT)-qPCR works well for quantitation of ASOs with simple modifications, but performs poorly with ASOs with complex modifications, like fully modified 2′-*O*-MOE [22]. Therefore, a chemical ligation qPCR method using chemical ligation between modified oligonucleotide probes, which have 3′-phosphorothioate and 5′-phenylsulfonyl groups, was developed [22]. However, this chemical ligation qPCR method requires oligonucleotide probes with modified 3′ and 5′ ends, which are neither commercially available nor easy to produce in most laboratories. A similar splint-ligation approach mediated by *Chlorella* virus SplintR DNA ligase (New England Biolabs, Ipswich, MA) has previously been reported to be used for miRNA quantification [23,24]. The SplintR qPCR assay is a quick and cost-effective method that can be used in any laboratory. In this study, we show that the SplintR qPCR assay effectively detects and quantifies ASOs in biological samples.

## Materials and Methods

### Synthesis of ASOs and ELISA probes

siRNA was purchased from Integrated DNA Technologies (IDT, Coralville, IA). Other chemically modified oligonucleotides (ASOs and ELISA probes) were synthesized in-house using Applied Biosystems 394 DNA/RNA synthesizers with standard detritylation and capping reagents, as described previously [25]. UnyLinker controlled-pore glass (CPG) supports (ChemGenes, Wilmington, MA) were used for ASOs and the capture probe and Phthalamide Amino C6 lcaa CPG supports (ChemGenes) were used for the detection probe. Activation involved the use of 5-benzylmercaptotetrazole (0.25 M in acetonitrile, ChemGenes), and oxidation using iodine (0.05 M in a 9:1 mixture of pyridine:water, ChemGenes), as well as sulfurization with 3-((dimethylamino-methylidene)amino)-3H-1,2,4-dithiazole-3-thione (DDTT) (0.1 M, ChemGenes). The DNA, 2′-OMe, 2′-*O*-MOE, LNA, and TFA-Amino C-6 CED phosphoramidites (ChemGenes) were dissolved in anhydrous acetonitrile to a final concentration of 0.15 M. The LNA-C phosphoramidite was dissolved in tetrahydrofuran:acetonitrile (v:v = 3:1). The 2′-OMe-U phosphoramidite was dissolved in acetonitrile:dimethylformamide (v:v = 8.5:1.5). Modified phosphoramidites were coupled for 10 min. The oligonucleotides were deprotected and cleaved from the CPG support by incubating with concentrated NH_4_OH at 55°C for 16 h.

Biotin and digoxigenin were incorporated through amide bond formation reaction between amino group and (+)-biotin *N*-hydroxysuccinimide ester (Sigma-Aldrich, St. Louis, MO) or Digoxigenin NHS-ester (Sigma-Aldrich). After incubating the mixture of amino-oligonucleotide (1 eq.) and NHS ester (15 eq.) in 200 mM 4-(2-hydroxyethyl)-1-piperazineethane sulfonic acid buffer [pH 8.4] for 48 h at 40°C, the reaction mixture was purified using reversed-phase high-performance liquid chromatography (Agilent, Santa Clara, CA). The oligonucleotides were desalted using Amicon Ultra Centrifugal Filters (Millipore, St. Louis, MO) or Glen Gel-Pak Desalting Columns (Glen Research, Sterling, VA). All ASOs and ELISA probes were characterized by electrospray ionization quadrupole time-of-flight LC-MS, using the negative ionization mode (Agilent).

### SplintR qPCR assay

The details of the probes and primers used in the SplintR qPCR assay reported here are as follows:

Probe pair 1

Probe A11: 5′ CTCGACCTCTCTATGGGCAGTCACGACAGGAGTCGCGCGC 3′

Probe B7: 5′ pTAGGGGCCGCTGAGTCGGAGACACGCAGGGCTTAA 3′

Probe pair 2

Probe A10: 5′ CTCGACCTCTCTATGGGCAGTCACGACAGGAGTCGCGCG 3′

Probe B8: 5′ pCTAGGGGCCGCTGAGTCGGAGACACGCAGGGCTTAA 3′

Probe pair 3

Probe A9: 5′ CTCGACCTCTCTATGGGCAGTCACGACAGGAGTCGCGC 3′

Probe B9: 5′ pGCTAGGGGCCGCTGAGTCGGAGACACGCAGGGCTTAA 3′

Probe pair 4

Probe A8: 5′ CTCGACCTCTCTATGGGCAGTCACGACAGGAGTCGCG 3′

Probe B10: 5′ pCGCTAGGGGCCGCTGAGTCGGAGACACGCAGGGCTTAA 3′

Probe pair 5

Probe A7: 5′ CTCGACCTCTCTATGGGCAGTCACGACAGGAGTCGC 3′

Probe B11: 5′ pGCGCTAGGGGCCGCTGAGTCGGAGACACGCAGGGCTTAA 3′

Forward primer: 5′ GCTCGACCTCTCTATGGGC 3′

Reverse primer: 5′ TTAAGCCCTGCGTGTCTCC 3′

Double-quenched probe: 5′ /FAM/CTAGCGCGC/ZEN/GACTCCGTCGTG/IABkFQ/ 3′

During careful checking at proof stage, it came to our awareness that the above double-quenched probe was missing a base. Please see Supporting Figure S6 for validation and comparison of the fully matched probe.

Probe B has a 5′ phosphate (p) required for enzymatic ligation to Probe A. The fluorescent dye used in this study was 6-carboxyfluorescein (FAM). ZEN was used as an internal quencher, while Iowa Black FQ (IABkFQ) was the 3′ quencher. Ligation probes, primers, and double-quenched probes used in the study were procured from IDT. The hybridization mixture was prepared as follows: 2 μL of the standard (10 μM to 10 fM) or the sample, 2 μL of 10 × SplintR ligase Reaction Buffer (New England Biolabs), 2 μL of probe A (100 nM), 2 μL of probe B (100 nM), and nuclease-free water (Thermo Fisher Scientific, Waltham, MA) up to 12 μL. In the case of standard for biological samples, 2 μL of serum or tissue lysate was added to the hybridization mixture to form the same matrix condition. The hybridization mixture was heated to 95°C for 5 min, and then cooled to 37°C at a rate of 0.1°C per second. After the hybridization of probes to the target oligonucleotides, 8 μL of diluted SplintR ligase (2.5 U/reaction, or up to 25 U/reaction for fully 2′-*O*-MOE-modified ASOs) was added to each hybridization mixture. Ligation of probes was achieved by incubation at 37°C for 30 min. The ligase mixture was heat-inactivated at 65°C for 20 min. In this study, if the probe was not specified then it designates that by default the probe pair 2 was used for the SplintR qPCR assay.

The qPCR reaction for quantification of ligated probes in the test article was prepared as follows: 2 μL of the ligation mixture described above, 10 μL of iTaq Super Mix (BioRad, Hercules, CA), 1 μL of forward and reverse primers (10 μM), 0.5 μL double-quenched probe (10 μM), and nuclease-free water added to make up a final volume of 20 μL. The qPCR reactions were conducted in triplicate using the CFX96 Real-time System (BioRad) under the cycling conditions: initial denaturation at 95°C for 3 min, followed by 40 cycles of denaturation at 95°C for 10 s and extension at 60°C for 30 s.

### 2′-*O*-MOE gapmer administration in mice

The administration of 2′-*O*-MOE gapmer was performed in female FVB mice (Charles River Laboratories, Wilmington, MA), 8 weeks of age, adhering to the Institutional Animal Care and Use Committee (IACUC) protocols of the University of Massachusetts Medical School (IACUC protocol A-2551). The 2′-*O*-MOE gapmer was diluted in 100 μL phosphate-buffered saline (PBS, Thermo Fisher Scientific) (200 μM), or 100 μL PBS for control group, and was administered by tail vein injection in five mice per group (treatment vs. control). After 1 h of the initial inoculation, mice were sacrificed. The blood and tissues of the euthanized animals were harvested.

### Preparation of serum and tissue samples

Blood was collected through cardiac puncture and allowed to stand for 1 h at room temperature for clotting without the addition of a clot activator. The blood sample was then centrifuged at 10,000 *g* for 5 min at room temperature. The serum was collected from the supernatant.

A sample of 20–30 mg of different tissues (liver, kidney, lung, heart, muscle, and brain) was removed from the mice treated with PBS or 2′-*O*-MOE gapmer, respectively, and weighed followed by tissue disruption using Tissuelyser II (Qiagen, Waltham, MA) in 30 μL mg^−1^ radioimmunoprecipitation assay buffer (Alfa Aesar, Haverhill, MA) containing 10 mM Tris-HCl [pH 7.4], 150 mM NaCl, 1% NP-40, 0.5% sodium deoxycholate, and 0.1% sodium dodecyl sulfate with 5 mM ethylenediaminetetraacetic acid (EDTA) and 1 mM ethyleneglycol-*bis*(β-aminoethylether)-*N*, *N*, *N*′, *N*′-tetraacetic acid (EGTA). Tissues were incubated for 1 h on ice with vortexing every 10 min, followed by centrifugation at 16,000 *g*, at 4°C for 30 min. The supernatant was collected into a new 1.5-mL microcentrifuge tube and stored at −80°C until subsequent analysis for the presence of 2′-*O*-MOE gapmer was performed.

### Advanced electrochemiluminescence-based hybridization ELISA

The probes and primers used in the advanced electrochemiluminescence (ECL)-based hybridization ELISA assay were as follows:

Capture probe: 5′ /Bio/G+AG+TCG+CG+CG 3′

Detection probe: 5′ +C+T+AGGGG+C/Dig/ 3′

The capture probe was conjugated to biotin (Bio) at the 5′ terminus, while the detection probe has digoxigenin conjugation (Dig) at the 3′ terminus, and LNA modified base is noted as +N.

We performed the hybridization ELISA assay as previously described by Thayer *et al.* [20]. Briefly, 5 μL of the standard (10 μM to 10 fM) was diluted in sample buffer (10 mM Tris-HCl [pH 8.0] and 1 mM EDTA) and final volume was adjusted to 50 μL. To simulate the condition in the biological matrix, 5 μL of serum was added during the dilution of the standard. To 50 μL of the 2 × mixture of probes (50 nM capture probe, 50 nM detection probe, 60 mM Na_2_PO_4_ [pH 7.0, dibasic], 1 M NaCl, 5 mM EDTA, and 0.02% Tween 20), the diluted standards were added and hybridized using the following incubation conditions: 95°C for 5 min, 40°C for 30 min, and hold at 12°C. After the hybridization, 95 μL of the hybridized standards was incubated in an MSD Gold 96-well Streptavidin SECTOR plate (Meso Scale Diagnostics, MSD, Rockville, MD) for 30 min with shaking. After incubation, the plate was washed thrice with 300 μL of 1 × KPL wash solution (SeraCare, Milford, MA) using a microplate washer (BioTek, Winooski, VT). After washing, the plates were incubated for 1 h with 50 μL of 0.5 μg mL^−1^ ruthenium-labeled anti-digoxigenin antibody (Roche, Mannheim, Germany), which was conjugated with ruthenium using the MSD GOLD SULFO-TAG NHS-Ester Conjugation kit (MSD), in SuperBlock T20 TBS Blocking Buffer (Thermo Fisher Scientific). After washing four times with 300 μL of 1 × KPL wash solution, 150 μL of 1 × MSD Read Buffer T (MSD) was added and ECL signal was measured using an ECL Imager (QuickPlex SQ 120, MSD).

### Data analysis

Mean, standard error of the mean (SEM), linear regression, and statistical tests were performed using GraphPad Prism V 8.4.2 (GraphPad Software, San Diego, CA). The regression lines for the SplintR qPCR assay and ELISA assay were calculated using the log of standard concentration in the sample vs. the mean quantification cycle (Cq) as well as the log of the mean ECL signal. The *p*-value in tissue quantification was calculated using the two-stage linear step-up procedure of Benjamini, Krieger, and Yekutieli with *Q* = 1%.

## Results and Discussion

**FIG. 1.**
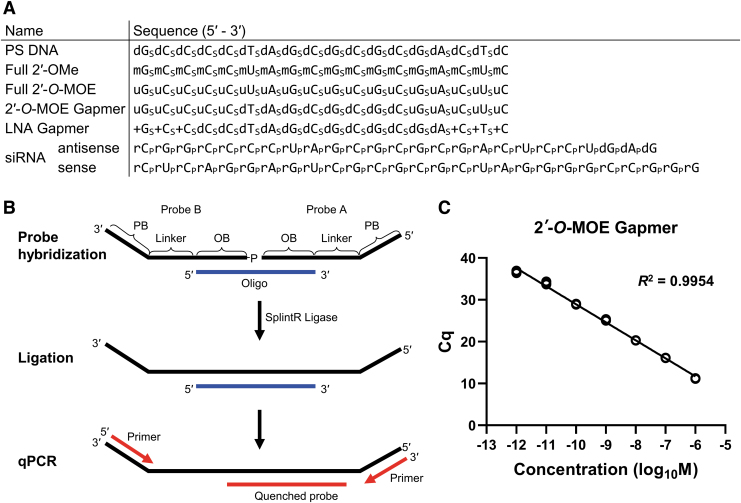
**(A)** Summary of ASOs used in this study. Oligonucleotide modifications have been represented as subscripts: p—phosphodiester linkage; s—phosphorothioate linkage; dN—DNA; mN—2′-OMe RNA; uN—2′-*O*-MOE RNA; +N—LNA; and rN—RNA. **(B)** Schematic diagram of ASO quantification using the SplintR qPCR assay. Probe A and B are hybridized to the target oligonucleotide by complementary OB sequence. After hybridization, two probes are ligated by SplintR ligase followed by qPCR with primers that recognize the PB sequence, and the amplification is detected using the quenched probe. **(C)** Quantification of the 2′-*O*-MOE gapmer dissolved in water. The qPCR for each standard point was performed in technical triplicate under the following cycling conditions: initial denaturation at 95°C for 3 min, followed by 40 cycles of denaturation at 95°C for 10 s and subsequent extension at 60°C for 30 s. Standard curve plotted from Cq value vs. the log of 2′-*O*-MOE gapmer concentration in the sample. 2′-*O*-MOE, 2′-*O*-methoxyethyl; ASOs, antisense oligonucleotides; Cq, quantification cycle; LNA, locked nucleic acid; OB, oligonucleotide binding; PB, primer binding; qPCR, quantitative real-time polymerase chain reaction.

Reliable and relatively straightforward bioanalytical assays for the detection and quantification of therapeutic ASOs will help in understanding the mechanism of action of oligonucleotides, which will facilitate their translation from preclinical to clinical settings. This study was aimed at developing a quick and reliable new method to detect and quantify ASOs in biological samples. Since enzymatic ligation with the *Chlorella* virus DNA ligase (SplintR ligase) [23,24] is a quick and cost-effective method to detect miRNAs, the objective of this study was to optimize and apply this enzymatic probe ligation-based qPCR technique to detect and quantify ASOs. The sequence and modification pattern of ASOs tested in this study are provided in Fig. 1A.

The quantification of ASOs was achieved using the SplintR qPCR assay. Each probe consisted of three parts: oligonucleotide-binding (OB), linker, and primer-binding (PB) regions. For hybridization, the OB region had a sequence complementary to the target ASO. The OB region was separated from the PB site by the linker region of the probe, thus extending the length of the ligation product for efficient PCR amplification. The PB regions were designed to be complementary to qPCR primers. The OB region varied depending on the target ASO, while the linker and PB regions remained constant. Probe A had a 3′-hydroxyl group, while probe B was synthesized with a 5′-phosphoryl group, which is essential for successful ligation. After probe A and B were hybridized to the target ASO, SplintR ligase was used to ligate the nick between probe A and B. As the SplintR ligase had a higher activity to the probe that was hybridized to the target ASO (compared with unhybridized probes), the ligated product increased proportionally with the amount of ASOs available in the sample. For specific and sensitive qPCR analysis, we designed a double-quenched qPCR probe, which relied on exonucleolytic release of a 5′ fluorophore **(**Fig. 1B**)**.

To determine whether the SplintR qPCR assay can be used to quantify modified ASOs, the 2′-*O*-MOE gapmer with a PS linkage was tested thereafter. We quantified a dilution series of 2′-*O*-MOE gapmer in water to generate the standard curves (Supplementary Fig. S1). The SplintR qPCR assay showed a broad range of linearity over seven orders of magnitude from 1 pM to 1 μM, with the coefficient of determination *R*^2^ > 0.99. The 2′-*O*-MOE gapmer ASO was detected at concentrations as low as 1 pM, demonstrating high sensitivity of the quantitative method described in this study (Fig. 1C).

To understand the scope of applications of the SplintR qPCR assay discussed in this study, we tested various types of ASOs, which were diluted in water for the study. This technique was tested for various types of ASOs with chemical modifications, including uniformly modified ASOs (PS DNA, 2′-OMe, and 2′-*O*-MOE), gapmers (2′-*O*-MOE and LNA), and siRNA, respectively. Our results yielded detection even for highly modified full 2′-*O*-MOE, although this substrate required a higher amount of SplintR ligase in the ligation step.

**FIG. 2.**
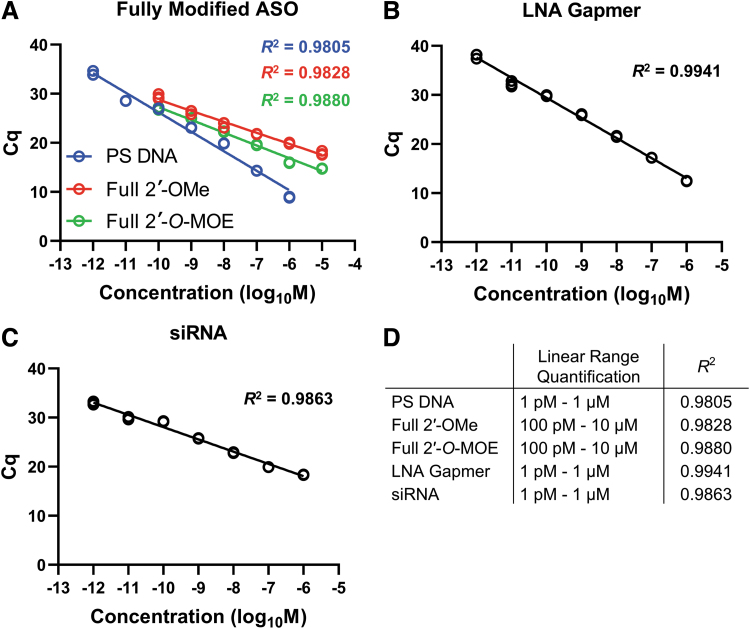
Application of the SplintR qPCR assay to quantify chemically and structurally diverse oligonucleotides. For quantification, each ASO was dissolved in water. The ASOs detected and quantified include **(A)** fully modified ASOs, **(B)** LNA gapmer, and **(C)** siRNA. Full 2′-*O*-MOE ASO used 25 U/reaction of SplintR ligase, while other ASOs used 2.5 U/reaction. The qPCR for each standard point was performed in technical triplicate. The standard curve was plotted as Cq value vs. the log of ASO concentration in the sample. **(D)** List of linear ranges of quantification and coefficients of determination (*R*^2^) for all the ASOs reported in this study.

Quantification assays performed for fully PS-modified DNA yielded positive detection outcomes across a linear range of seven orders of magnitude from 1 pM to 1 μM. In contrast, the fully modified 2′-OMe and 2′-*O*-MOE ASOs were detected over 6 orders of magnitude from 100 pM to 10 μM (Fig. 2A). To obtain a linear range of detection of the fully modified 2′-*O*-MOE ASO a 10-fold higher amount of ligase (25 U/reaction) was required because the amount of ligase used for detection of other ASOs (2.5 U/reaction) failed to achieve a broad range of linearity in case of fully modified 2′-*O*-MOE ASO (Supplementary Fig. S2). The amount of ligase (25 U/reaction) used for detection of the fully modified 2′-*O*-MOE ASO was optimized based on standardization experiments conducted as a part of this study. In addition, the sensitivity and linear range quantification were different for different pairs of ligation probe sequences (Supplementary Fig. S3). The bulky modification of 2′-*O*-MOE RNA seemed to partially inhibit the enzymatic ligation reaction, requiring some optimization of both ligation probe sequences and reaction conditions.

Next, we tested a LNA gapmer widely used for RNase H-mediated gene silencing along with a 2′-*O*-MOE gapmer. The LNA gapmer showed optimum quantification over seven orders of magnitude from 1 pM to 1 μM (Fig. 2B), similar to that obtained for 2′-*O*-MOE gapmer. We also tested siRNA, which resulted in the linear range quantification over 7 orders of magnitude from 1 pM to 1 μM (Fig. 2C). The specifics of the linear range quantification and the coefficient of determination (*R*^2^) has been presented in Fig. 2D.

Preliminary tests to evaluate the efficacy of the SplintR qPCR assay to quantify ASOs in biological samples involved preparation of a dilution series of 2′-*O*-MOE gapmer in mouse serum and mouse liver lysate, respectively, which was followed by a subsequent round of the SplintR qPCR assay. Our results show that the SplintR qPCR assay allowed efficient and sensitive detection of 2′-*O*-MOE gapmer at low concentrations ranging from 1 pM to 1 μM in serum and liver lysate-based samples, with the coefficient of determination *R*^2^ > 0.99 (Supplementary Fig. S4).

**FIG. 3.**
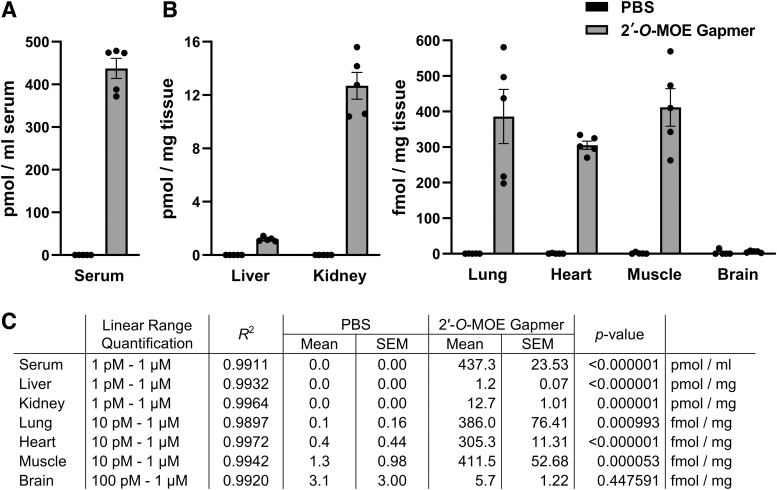
Detection of 2′-*O*-MOE gapmer after tail vein injection in a mouse model. Blood and tissues were collected 1 h after administration of 20 nmol 2′-*O*-MOE gapmer or PBS (control). Serum was separated from blood, and tissues were lysed using RIPA buffer. **(A, B)** The concentration of 2′-*O*-MOE gapmer in **(A)** serum and **(B)** tissues was quantified using the SplintR qPCR assay. *Bars* represent mean ± SEM from five animals with values of the individual animals (●); each data point represents the average of technical triplicate qPCR wells. **(C)** The table lists the linear range of the standard curve and the concentration of the 2′-*O*-MOE gapmer in tissue samples. The linear range of qualification and the coefficient of determination (*R*^2^) were obtained from standard curves, which have been presented in Supplementary Fig. S5. The *p*-value was calculated by comparing the data from each 2′-*O*-MOE gapmer-treated group to the corresponding PBS (control) group using a *t*-test. PBS, phosphate-buffered saline; RIPA, radioimmunoprecipitation assay; SEM, standard error of the mean.

In the subsequent experiments, the efficiency of the SplintR qPCR assay in quantifying the ASOs administered to mice through tail vein injection was determined. The test animals were administered with 2′-*O*-MOE gapmer (20 nmol) by tail vein injection. After 1 h, the animals were euthanized and serum, liver, kidney, lung, heart, muscle, and brain samples from each mouse were collected for subsequent quantification of 2′-*O*-MOE gapmer using the SplintR qPCR assay (Fig. 3). Negative controls included samples from mice injected with PBS alone. We generated standard curves for 2′-*O*-MOE gapmer diluted in serum or tissue lysates: serum, liver, and kidney with linear detection across seven orders of magnitude ranging from 1 pM to 1 μM, and lung, heart, and muscle, with linear detection across six orders of magnitude ranging from 10 pM to 1 μM (Supplementary Fig. S5). The SplintR qPCR assay could only detect the 2′-*O*-MOE gapmer in brain lysate across five orders of magnitude from 100 pM to 1 μM (Supplementary Fig. S5). The assay has reduced sensitivity for brain lysate samples, which may be attributed to the high lipid content of the brain tissue. Nevertheless, the results presented in this study indicate that the SplintR qPCR assay is compatible with biological samples and efficiently detects ASOs. The SplintR qPCR assay revealed that the 2′-*O*-MOE gapmer levels were abundant in serum (437.3 pmol mL^−1^), liver (1.2 pmol mg^−1^), and kidney (12.7 pmol mg^−1^), respectively. Lower levels were detected in lung (386.0 fmol mg^−1^), heart (305.3 fmol mg^−1^), and muscle (411.5 fmol mg^−1^), respectively. The quantification assay in brain tissue showed no detection above background levels, which may be attributed to minimal transfer across the blood–brain barrier.

**FIG. 4.**
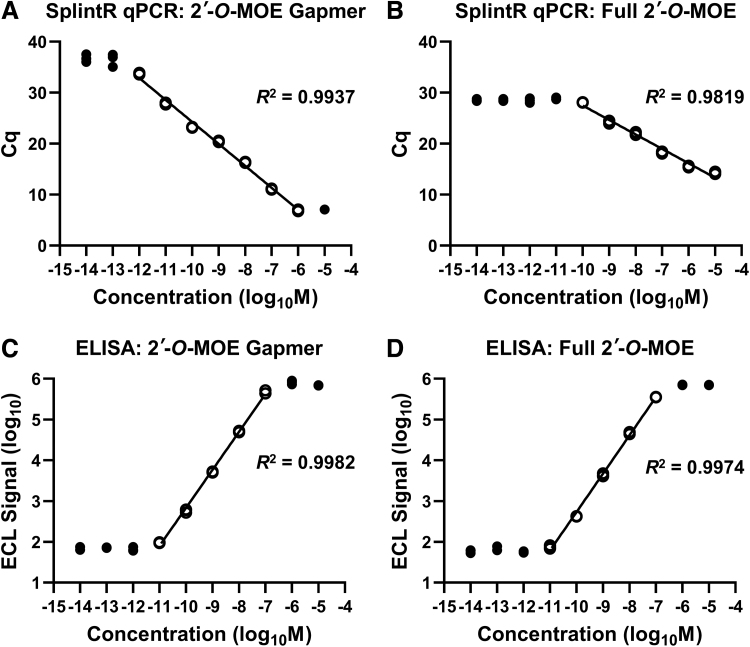
Comparison between the SplintR qPCR assay and ELISA method. The ASOs were detected in serum-supplemented condition. Each standard point was performed in technical triplicate. Values outside the linear range have been represented with a *black circle* (●). SplintR qPCR assay detection of **(A)** 2′-*O*-MOE gapmer and **(B)** full 2′-*O*-MOE ASO, using 2.5 U/reaction and 25 U/reaction SplintR ligase, respectively. Standard curve plotted from Cq value vs. the log of ASO concentration in the sample. ELISA detection of **(C)** 2′-*O*-MOE gapmer and **(D)** full 2′-*O*-MOE ASO. Standard curve plotted from the log of ECL signal vs. the log of ASO concentration in the sample. ECL, electrochemiluminescence; ELISA, enzyme-linked immunosorbent assay.

For a head-to-head comparison of the SplintR qPCR assay with the more commonly used hybridization ELISA method, the 2′-*O*-MOE gapmer and the fully modified 2′-*O*-MOE ASO were tested in both assays under serum matrix-based conditions. We used a highly sensitive ELISA assay based on ECL detection [20]. In case of the 2′-*O*-MOE gapmer, the SplintR qPCR assay had a broader detection range, extending the linear detection range to both 10-fold higher and 10-fold lower concentrations relative to that of the ELISA assay (Fig. 4A, C). In contrast, for the fully modified 2′-*O*-MOE ASO, the SplintR qPCR assay showed 10-fold less sensitivity compared to the ELISA assay (Fig. 4B, D). Nevertheless, it exhibited a broad range of linearity. Thus, compared to the SplintR qPCR assay, the ELISA assay was more tolerant to bulky modifications of the 2′-*O*-MOE RNA, perhaps because the former relies on hybridization and is not dependent on the enzymatic ligation of the probes.

To improve platform technologies for oligonucleotide therapeutics and design apply them to new diseases, quick and cost-effective techniques to detect and quantify therapeutic ASOs are pertinent. The SplintR qPCR assay does not require specialized equipment, but uses qPCR reagents and equipment available to most molecular biology laboratories. The SplintR qPCR assay has several advantages over the previously described chemical ligation qPCR strategy [22]. The *Chlorella* virus SplintR ligase is readily available and can be used directly without any special modifications of the splint ligation probes, and the procedure is faster. Thus, the SplintR qPCR assay is a simple and cost-effective way to detect and quantify therapeutic ASOs in biological samples, such as serum or biopsy-based tissues. The dynamic range of the assay is superior to that of a gold-standard hybridization ELISA method even with ECL detection, and will be easier to implement for many laboratories. Therefore, the SplintR qPCR assay described in this study is likely to become a useful tool for groups studying therapeutic development and pharmacokinetics of oligonucleotide therapeutics.

## Supplementary Material

Supplemental data

Supplemental data

Supplemental data

Supplemental data

Supplemental data

Supplemental data
