# Supplementary material for: Quantification of Antisense Oligonucleotides by Splint Ligation and Quantitative Polymerase Chain Reaction
Source: Nucleic Acid Ther. 2022 Jan 31;32(1):66–73. doi: 10.1089/nat.2021.0040 (PMC8817697; doi:10.1089/nat.2021.0040)
Supplement: Supplemental data [file Supp_FigS1.pdf]

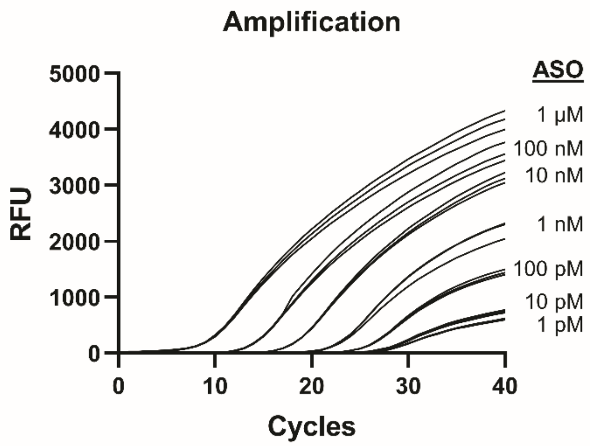

Supplementary Figure S1. Quantification of 2′-*O*-MOE gapmer using the SplintR qPCR assay. Amplification curve of a 2′-*O*-MOE gapmer serially diluted in water for a concentration range of 1 pM to 1 µM. The concentration of the 2′-*O*-MOE gapmer is mentioned at the right of the amplification curve. The qPCR for each concentration was performed in technical triplicate, with each curve shown above. RFU represents relative fluorescence units.
