# Supplementary material for: Quantification of Antisense Oligonucleotides by Splint Ligation and Quantitative Polymerase Chain Reaction
Source: Nucleic Acid Ther. 2022 Jan 31;32(1):66–73. doi: 10.1089/nat.2021.0040 (PMC8817697; doi:10.1089/nat.2021.0040)
Supplement: Supplemental data [file Supp_FigS2.pdf]

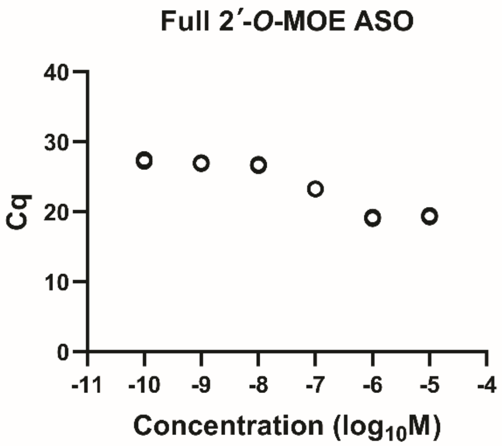

Supplementary Figure S2. Standard curve for fully modified 2′-*O*-MOE ASO diluted in water using 2.5 U/reaction of SplintR ligase. The qPCR for each standard point was performed in technical triplicate.
