# Supplementary material for: Quantification of Antisense Oligonucleotides by Splint Ligation and Quantitative Polymerase Chain Reaction
Source: Nucleic Acid Ther. 2022 Jan 31;32(1):66–73. doi: 10.1089/nat.2021.0040 (PMC8817697; doi:10.1089/nat.2021.0040)
Supplement: Supplemental data [file Supp_FigS3.pdf]

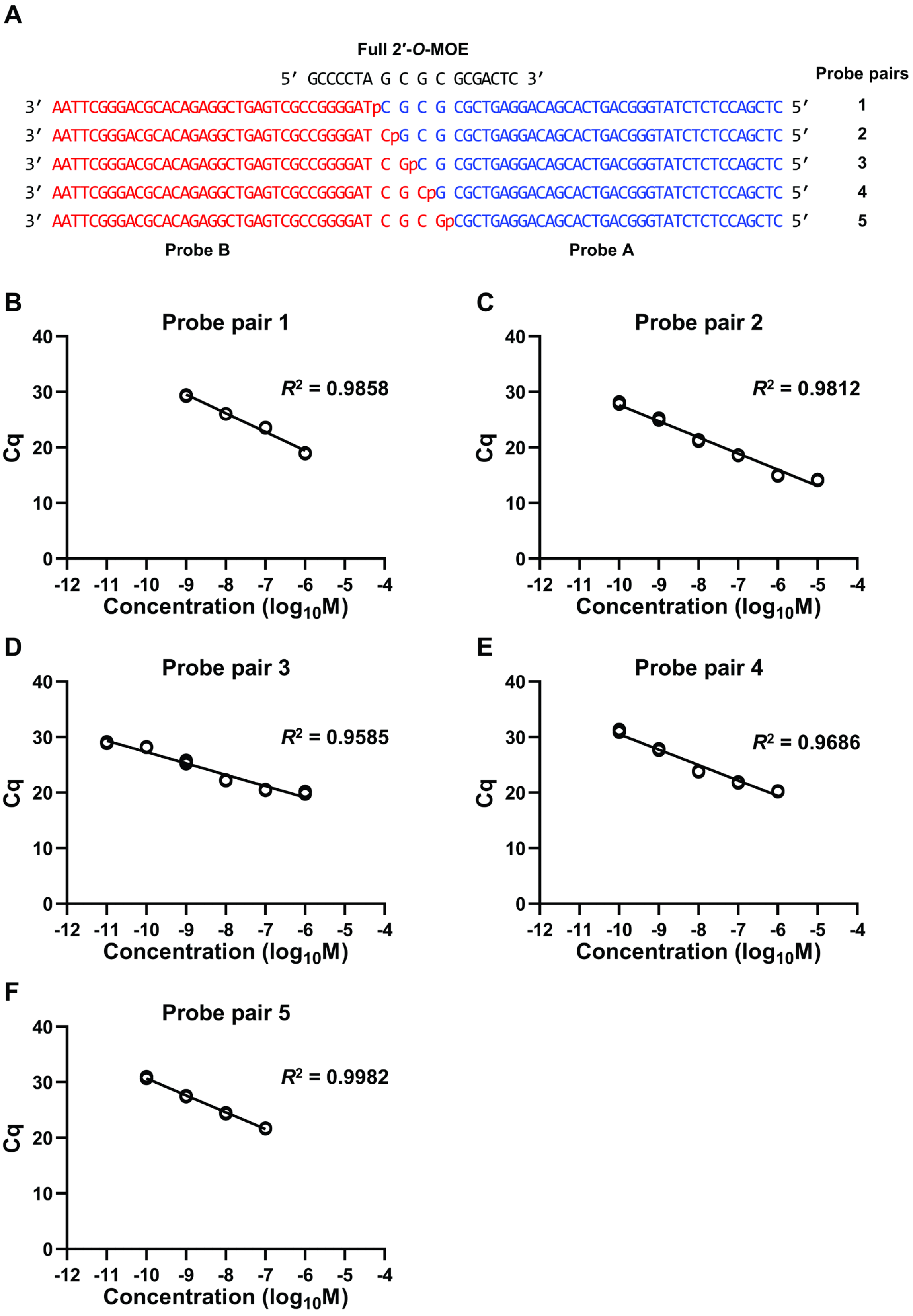
A
Full 2′-O-MOE
5′ GCCCCTA G C G C GCGACTC 3′
Probe pairs
3′ AATTCGGGACGCACAGAGGCTGAGTCGCCGGGGATpC G C G CGCTGAGGACAGCACTGACGGGTATCTCTCCAGCTC 5′ 1
3′ AATTCGGGACGCACAGAGGCTGAGTCGCCGGGGAT CpG C G CGCTGAGGACAGCACTGACGGGTATCTCTCCAGCTC 5′ 2
3′ AATTCGGGACGCACAGAGGCTGAGTCGCCGGGGAT C GpC G CGCTGAGGACAGCACTGACGGGTATCTCTCCAGCTC 5′ 3
3′ AATTCGGGACGCACAGAGGCTGAGTCGCCGGGGAT C G CpG CGCTGAGGACAGCACTGACGGGTATCTCTCCAGCTC 5′ 4
3′ AATTCGGGACGCACAGAGGCTGAGTCGCCGGGGAT C G C GpCGCTGAGGACAGCACTGACGGGTATCTCTCCAGCTC 5′ 5
Probe B
Probe A
B
Probe pair 1
R2 = 0.9858
C
Probe pair 2
R2 = 0.9812
D
Probe pair 3
R2 = 0.9585
E
Probe pair 4
R2 = 0.9686
F
Probe pair 5
R2 = 0.9982
Cq
Concentration (log10M)

Supplementary Figure S3. Optimization of ligation probe sequences for fully modified 2′-*O*-MOE ASO. **(A)** Details of the pairs of ligation probe A (in blue) and probe B (in red) with different complementary sequences are presented along with the target ASO sequence (in black). **(B-F)** Standard curve for fully modified 2′-*O*-MOE ASO diluted in water using 25 U/reaction of SplintR ligase by different ligation probe pair. The qPCR for each standard point was performed in technical triplicate.
