# Supplementary material for: Quantification of Antisense Oligonucleotides by Splint Ligation and Quantitative Polymerase Chain Reaction
Source: Nucleic Acid Ther. 2022 Jan 31;32(1):66–73. doi: 10.1089/nat.2021.0040 (PMC8817697; doi:10.1089/nat.2021.0040)
Supplement: Supplemental data [file Supp_FigS4.pdf]

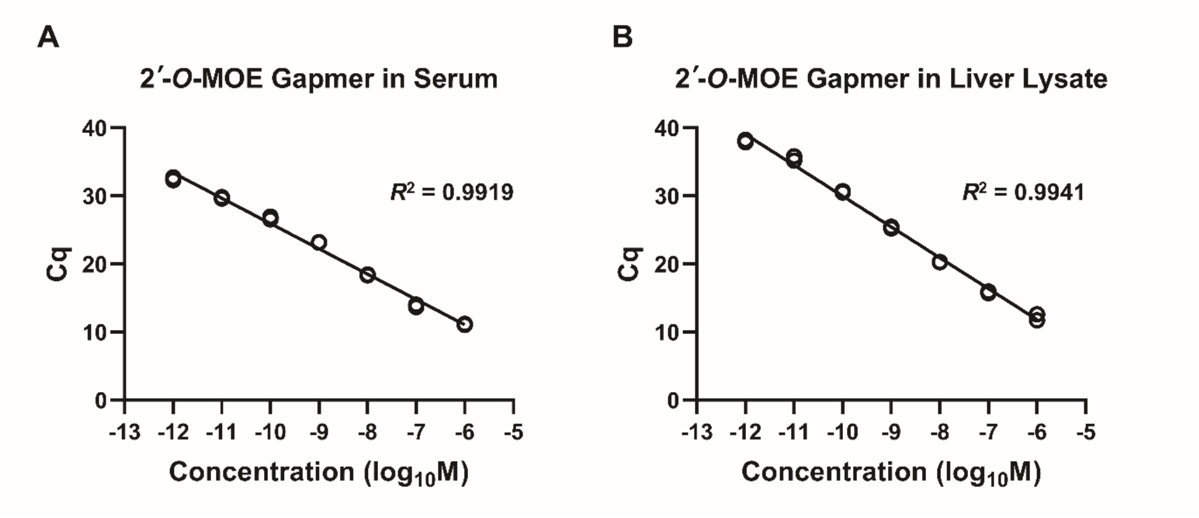

Supplementary Figure S4. Evaluation of the efficacy of the SplintR qPCR assay to quantify ASOs in biological matrices. The standard curve was generated using diluted 2′-*O*-MOE gapmer in a matrix containing mouse **(A)** serum or **(B)** liver lysate. The qPCR for each standard point was performed in technical triplicate.
