# Supplementary material for: Quantification of Antisense Oligonucleotides by Splint Ligation and Quantitative Polymerase Chain Reaction
Source: Nucleic Acid Ther. 2022 Jan 31;32(1):66–73. doi: 10.1089/nat.2021.0040 (PMC8817697; doi:10.1089/nat.2021.0040)
Supplement: Supplemental data [file Supp_FigS5.pdf]

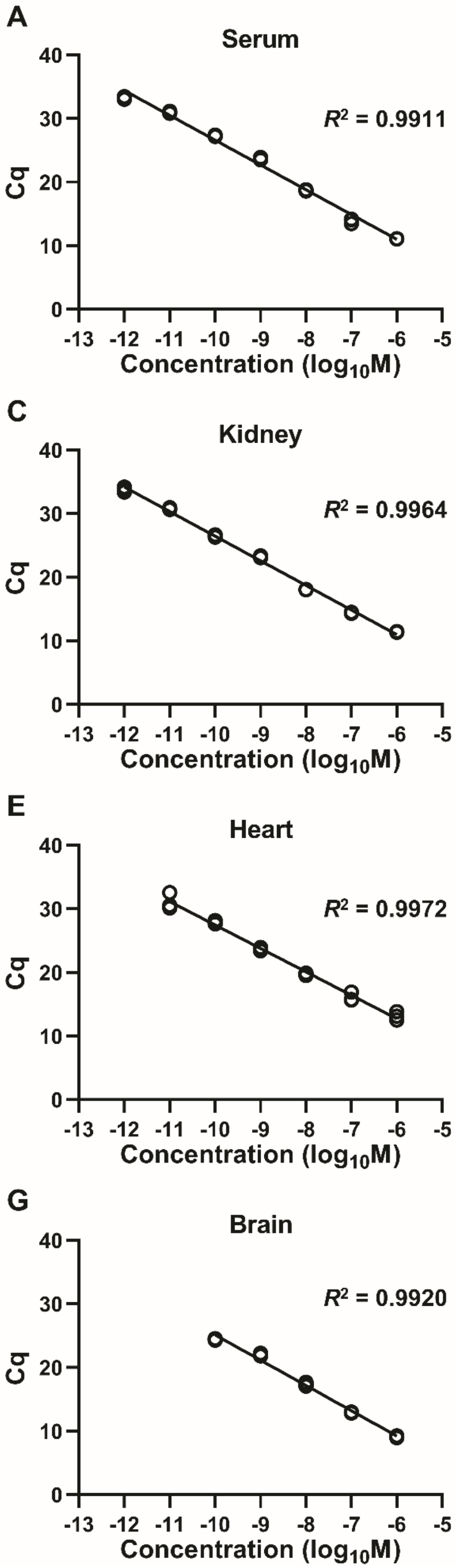

A
Serum
R^2 = 0.9911
B
Liver
R^2 = 0.9932
C
Kidney
R^2 = 0.9964
D
Lung
R^2 = 0.9897
E
Heart
R^2 = 0.9972
F
Muscle
R^2 = 0.9942
G
Brain
R^2 = 0.9920
Cq
Concentration (log_{10}M)

Supplementary Figure S5. Standard curve for 2′-*O*-MOE gapmer from: **(A)** serum, and **(B-G)** different tissue lysates. The linear regression obtained from each biological matrix was used for quantification of 2′-*O*-MOE gapmer in Figure 3. The qPCR for each standard point was performed in technical triplicate.
