# Supplementary material for: Quantification of Antisense Oligonucleotides by Splint Ligation and Quantitative Polymerase Chain Reaction
Source: Nucleic Acid Ther. 2022 Jan 31;32(1):66–73. doi: 10.1089/nat.2021.0040 (PMC8817697; doi:10.1089/nat.2021.0040)
Supplement: Supplemental data [file Supp_FigS6.pdf]

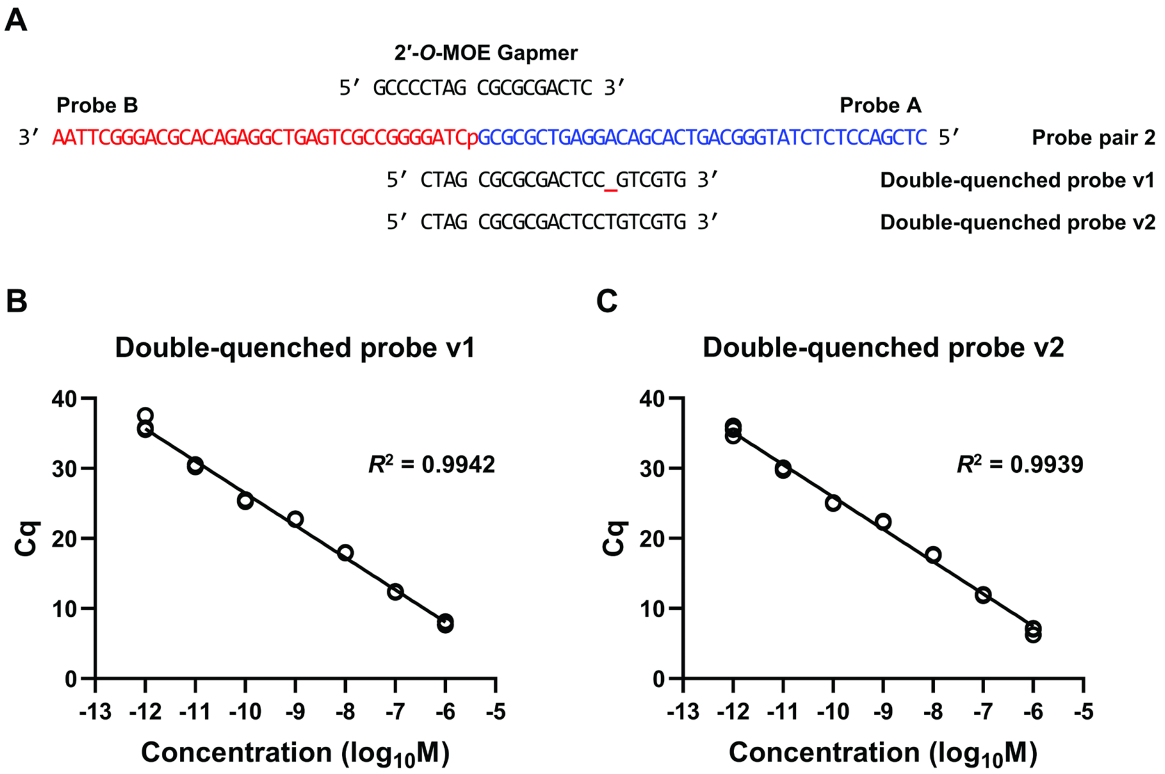

Supplementary Figure S6. During revision of the manuscript, we became aware that this work was completed with one base inadvertently omitted from the double-quenched probe (v1 shown above). Therefore, we purchased a fully matched double-quenched probe (v2 shown above) to verify whether this had any effect on qPCR sensitivity. **(A)** Sequences of the pair of ligation probe A (in blue) and probe B (in red) are presented along with the target ASO sequence and double-quenched probes (in black). The double-quenched probe v1 has omitted one complementary base (red underline) while the double-quenched probe v2 is fully matched to the ligated probe sequence. **(B, C)** Standard curves were obtained for 2′-*O*-MOE gapmer diluted in mouse serum by the SplintR qPCR assay with the double-quenched probe v1 **(B)** and the double-quenched probe v2 **(C)**. The qPCR for each standard point was performed in technical triplicate. The two probes gave very similar performance, although the fully matched probe gave slightly higher sensitivity (slightly lower Cq values).
